# Supplementary material for: Peptide fusion improves prime editing efficiency
Source: Nat Commun. 2022 Jun 18;13:3512. doi: 10.1038/s41467-022-31270-y (PMC9206660; doi:10.1038/s41467-022-31270-y)
Supplement: Supplementary file 1 — Supplementary Information [file 41467_2022_31270_MOESM1_ESM.pdf]

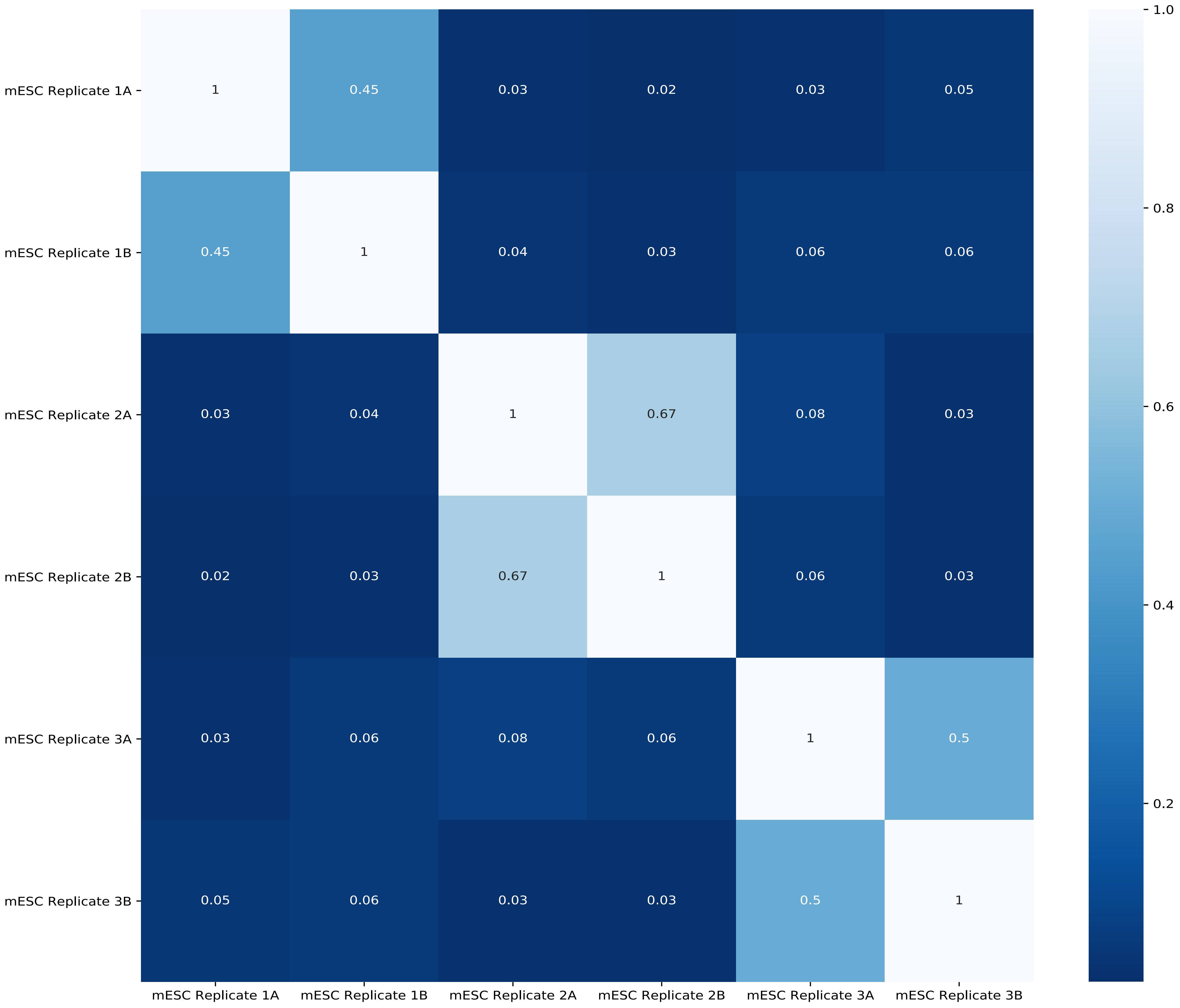

Supplementary Figure 1. Replicate consistency in 12,000-peptide PepSEq screens.
(a) Comparison of prime editing efficiency in 12,000-peptide PepSEq screens for all pairs of replicates with independent and shared peptide-PE2 integration. Technical replicates were combined and biological replicates were used for analysis.

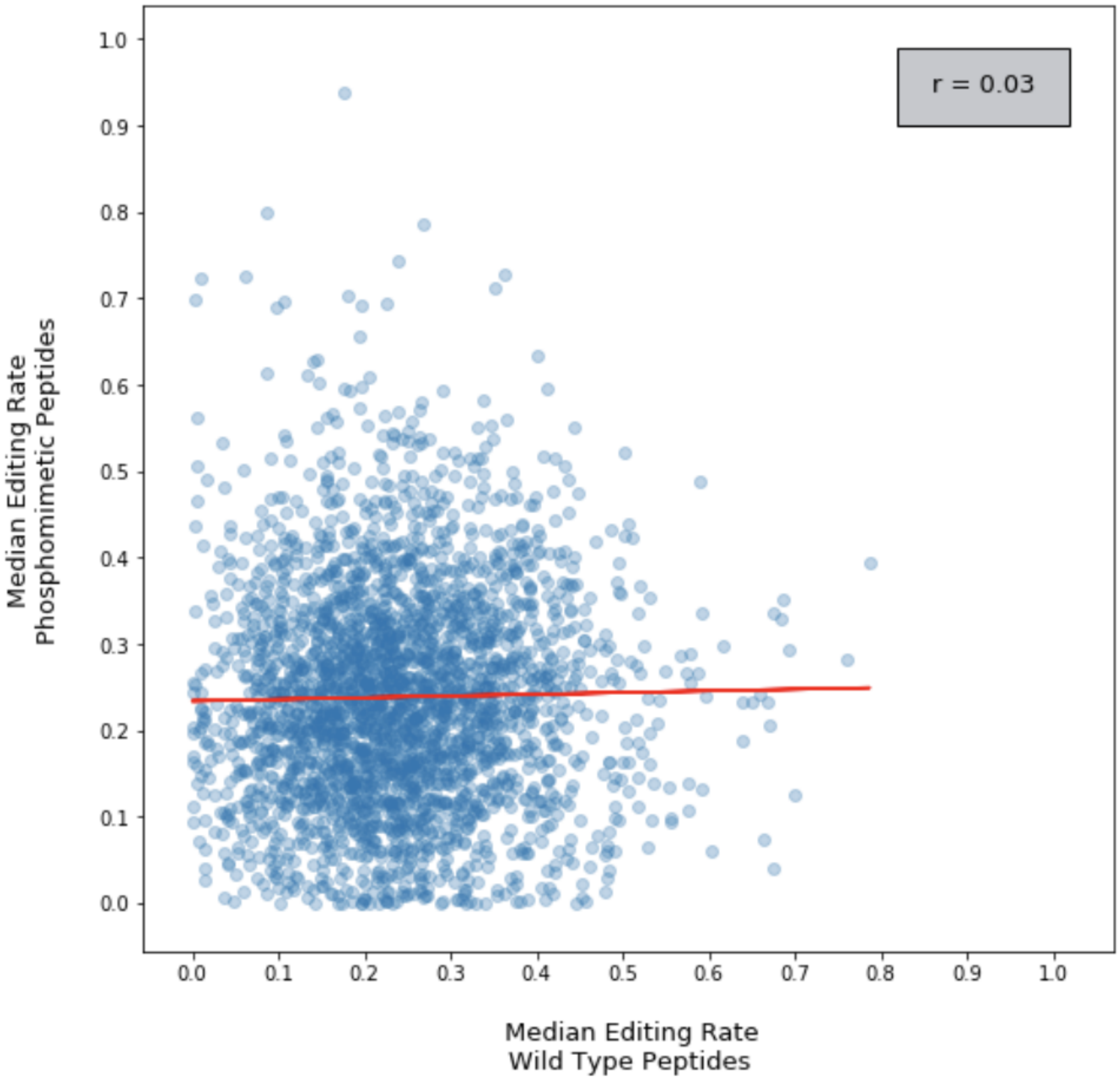

Supplementary Figure 2: Phosphomimetic and WT peptide pair editing rates.
Distributions of phosphomimetic and wild type peptides pairs. Editing rates among peptides in phosphomimetic and wild type configurations. Each peptide is measured as the median of three replicates when excluding instances of counts of 0. We find little correlation in editing rates between peptides of the two configurations (Spearman r = 0.03).

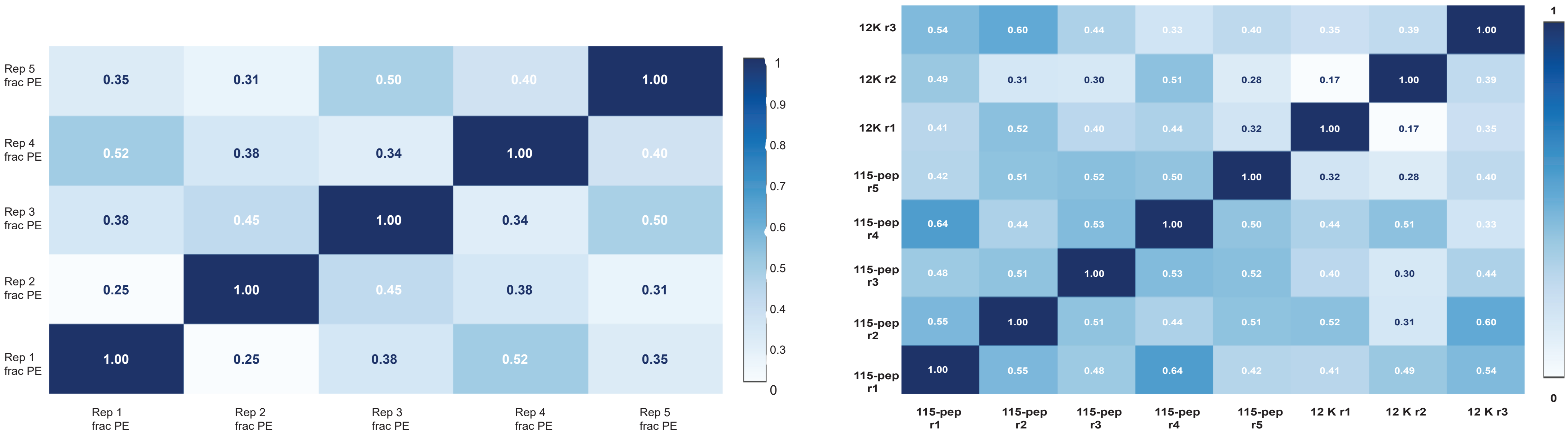

Supplementary Figure 3: A 115-peptide PepSEq screen reveals peptides that induce replicate-consistent increases in prime editing efficiency.

(a) Correlation table of prime editing efficiency in five biological replicate 115-peptide PepSEq screens.

(b) Correlation table of prime editing efficiency of 45 strongest prime editing-enhancing peptides and 10 control peptides in five biological replicate 115-peptide PepSEq screens and three biological replicate 12,000-peptide screens.

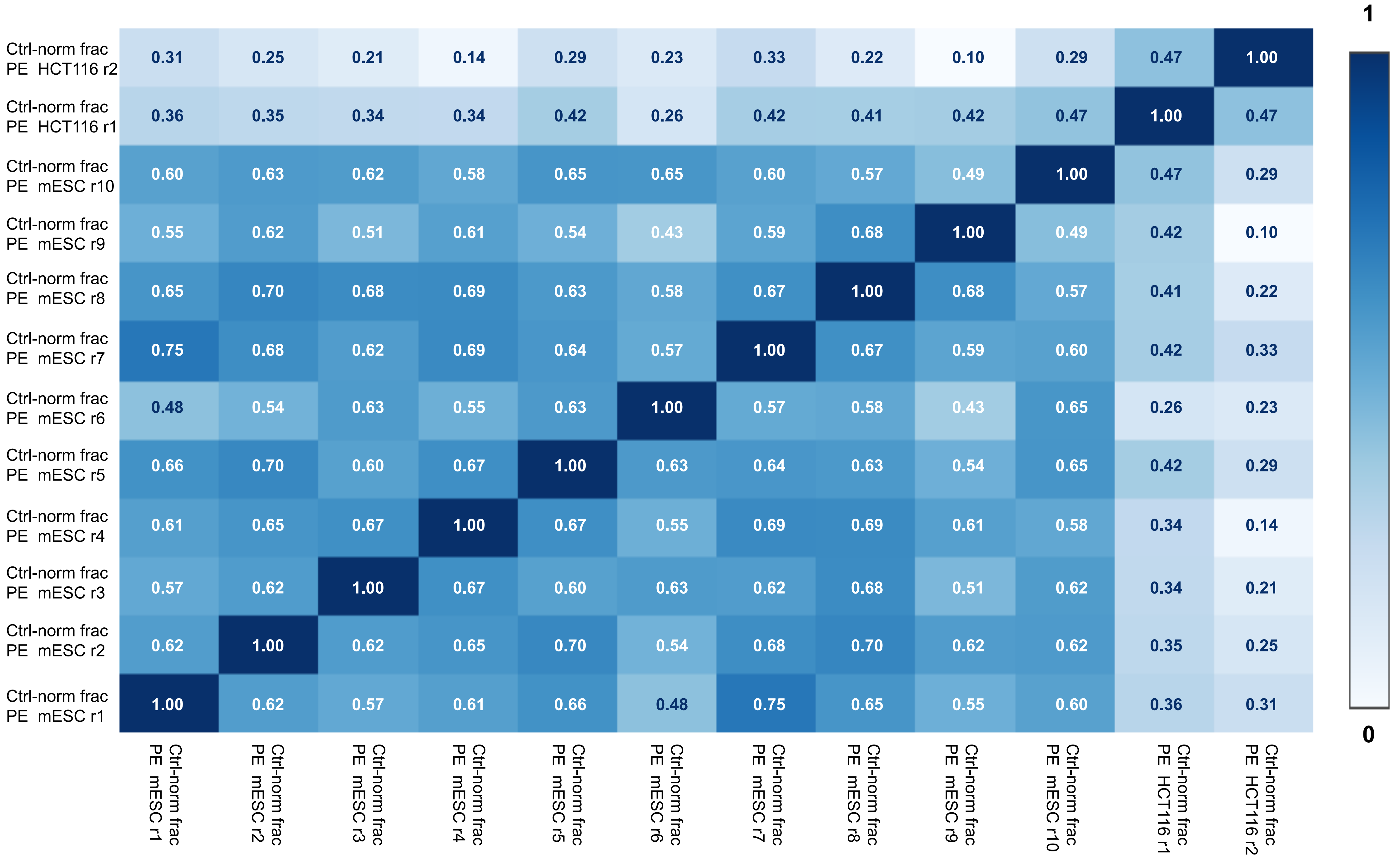

Supplementary Figure 4: Correlation table of prime editing efficiency in 10 biological replicate dual-peptide PepSEq screens in mESC and two biological replicates in HCT-116.

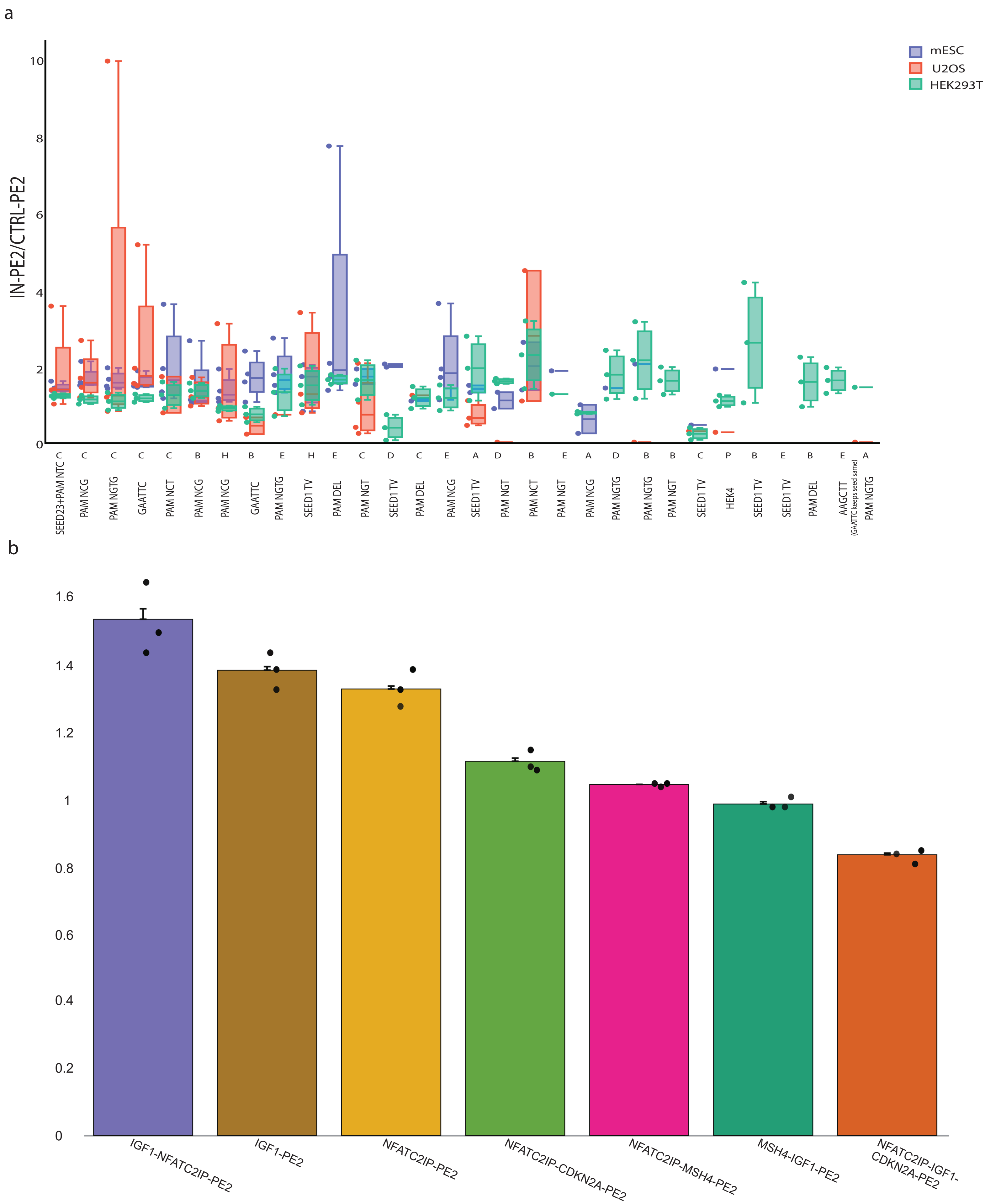

Supplementary Figure 5: A dual-peptide PepSEq screen reveals linear contribution of prime editing-enhancing peptides.

(a) Comparison of control-normalized prime edited fraction for 100 dual-peptide pairs in mESC, u2os and Hek293FT cells using IN-PE2. Data shown for top 30 edited sites of 100-site library. n=4 independent biological replicates per cell line. Boxes represent the 25–75 percentile ranges with the median of horizontal line. The ends of vertical lines represent minimum or maximum values. The upper and lower whiskers represent scores outside the middle 50%. Source data and exact p-values are provided as a Source Data file.

(b) PE effect of each dipeptide from dual-peptide screen in 100-site library
Data and error bars in b indicate the median and standard deviation of 3 independent biological replicates.

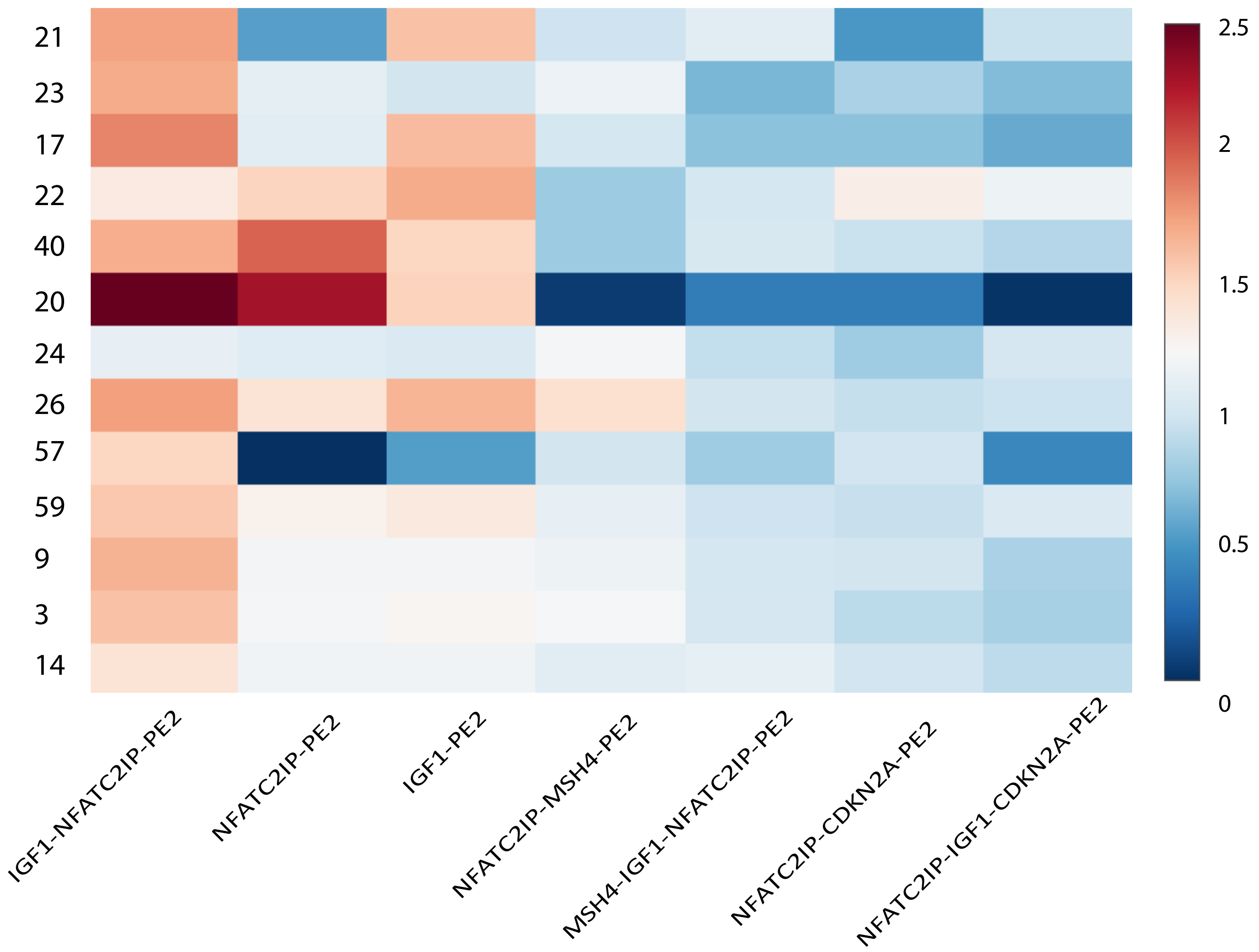

Supplementary Figure 6: Peptide-PE2 editing in a 100-member pegRNA-target library. Heat map of control-normalized mESC prime editing efficiency at the 13 sites within the 100-site library with >0.5% editing in control PE2-treated cells. Peptides show consistent effects on prime editing across target sites in several cell lines (HEK293FT, HCT-116, U2OS). (n=4 independent replicates). Numbers on X axis correspond to targets of 100-site library. (see supplementary Table 5 for oligo sequences)

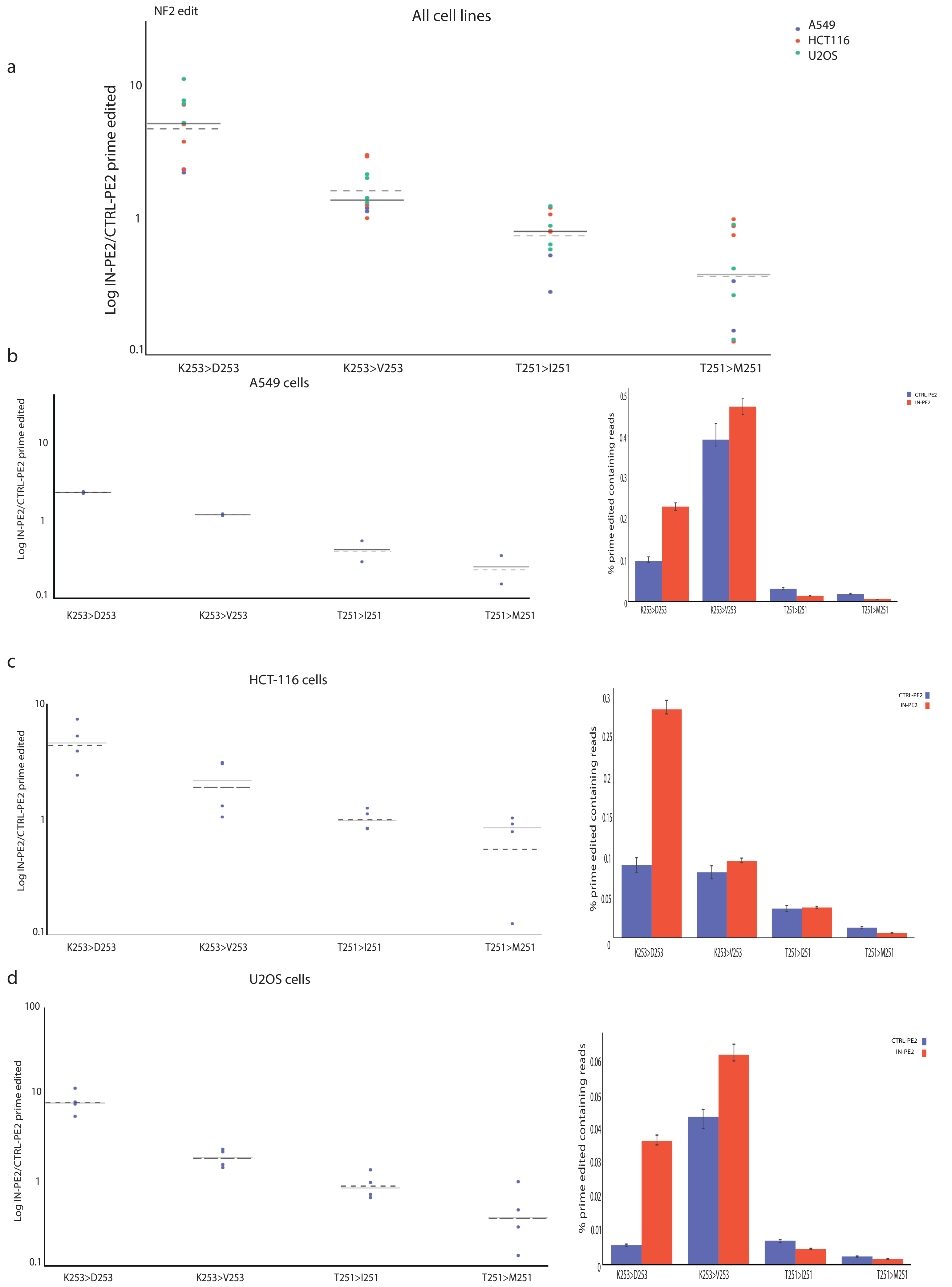

Supplementary Figure 7: Six endogenous test sites at NF2 locus in 3 cell lines (U2OS, A549, and HCT-116). Figure panels on the left present CTRL-PE2-normalized prime editing with IN-PE2. Figure panels on the right show percentage of editing for each SNP. Data and error bars indicate the median and standard deviation of three independent biological replicates per each cell line.

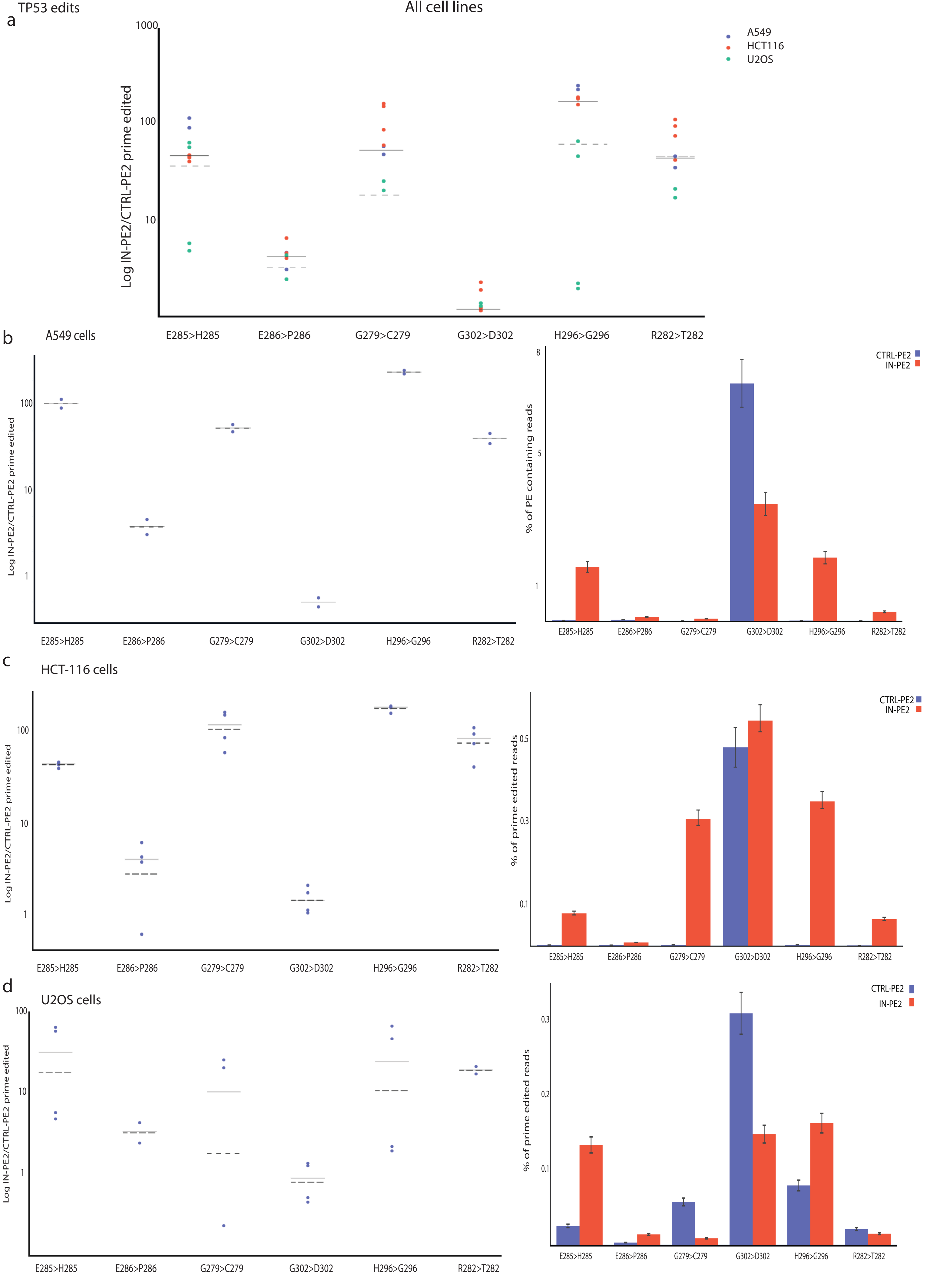

Supplementary Figure 8: Six endogenous test sites at TP53 locus in 3 cell lines (U2OS, A549, and HCT-116).
Figure panels on the left present CTRL-PE2-normalized prime editing with IN-PE2. Figure panels on the right show percentage of editing for each SNP. Data and error bars indicate the median and standard deviation of three independent biological replicates per each cell line.

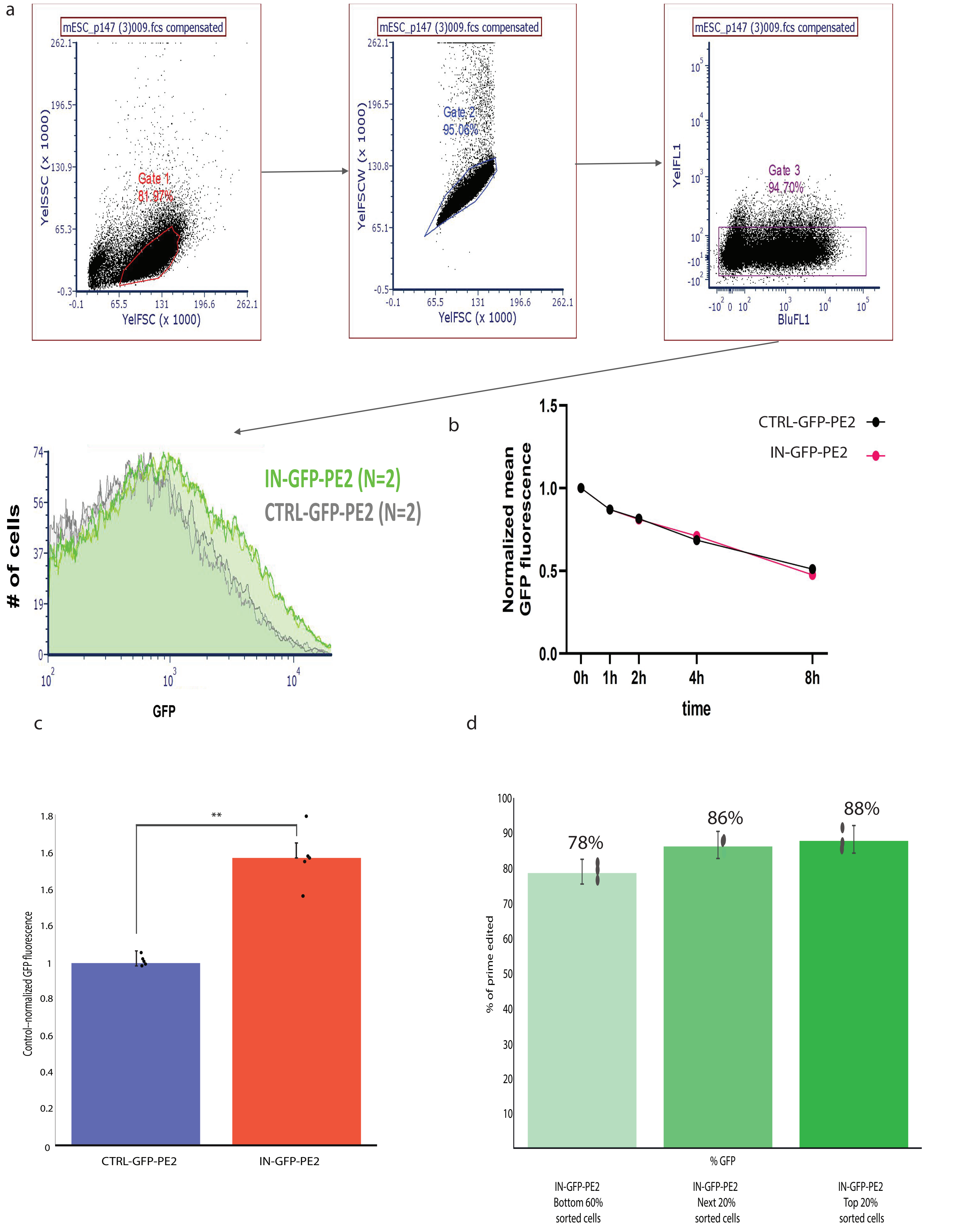

Supplementary Figure 9: IN-PE2 is expressed at an increased level as compared to CTRL-PE2.

(a) Full gating for flow cytometric analysis of IN-GFP-PE2 and CTRL-GFP-PE2.

(b) IN-GFP-PE2 and CTRL-GFP-PE2 fluorescence during an 8-hour cycloheximide timecourse, normalized to fluorescence in untreated cells.

(c) IN-GFP-PE2 shows higher GFP signal than CTRL-GFP-PE2 in mESC. Data and error bars indicate the median and standard deviation of five independent biological replicates. Statistically significant differences are denoted as follows: $*p < 0.05$, $**p < 0.01$, $***p < 0.001$

(d) IN-GFP-PE2 mESC cells were sorted into 3 different populations based on GFP fluorescence (Top 20%, next 20% and bottom 60%) and their prime editing yields were measured via NGS. (n=3 independent biological replicates)

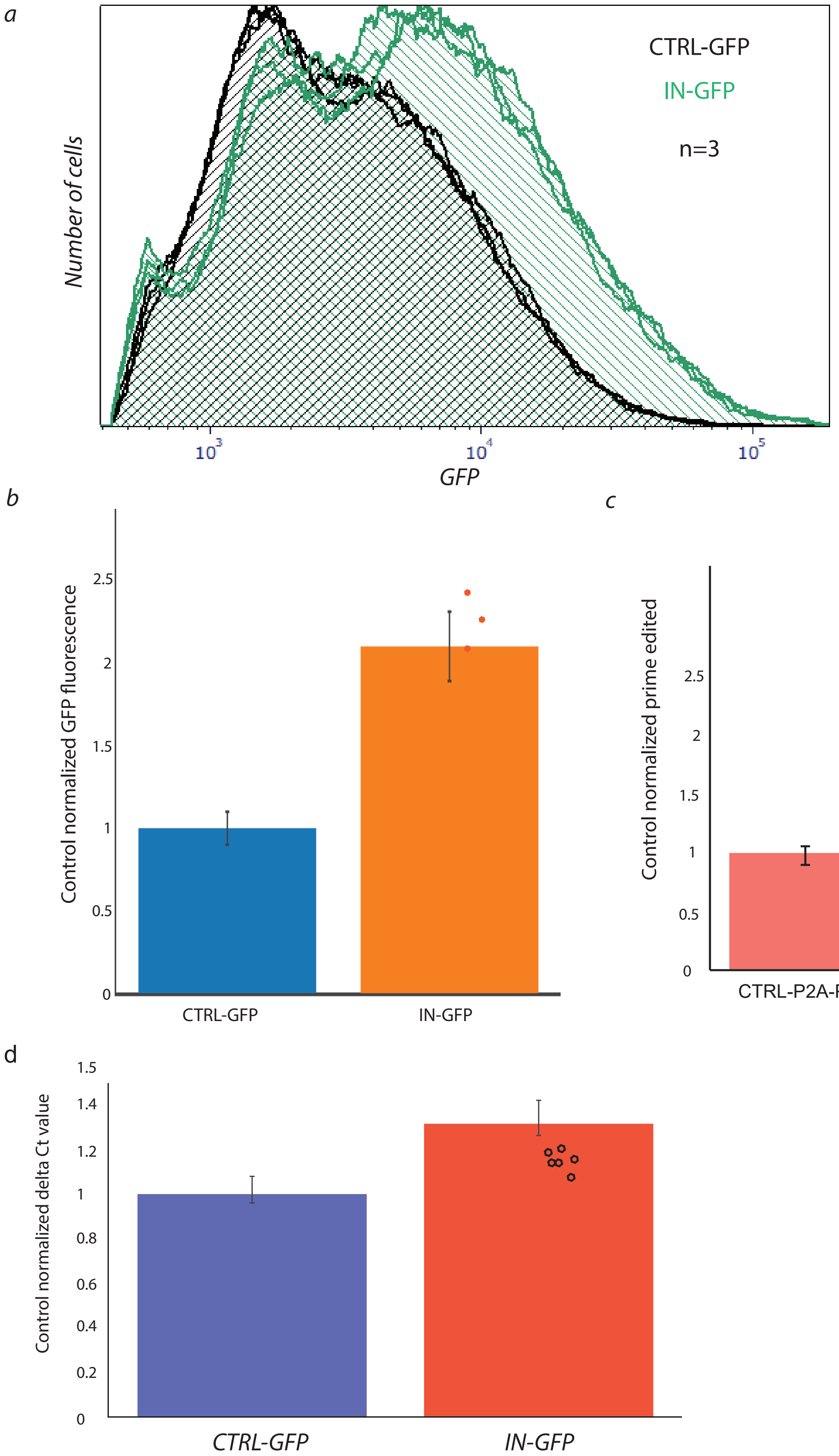

Supplementary Figure 10: IN dipeptide increases levels of PE2 within the cells

a) IN dipeptides fused to GFP show robust increase in cellular GFP protein levels. (n= 3 biological replicates of mESC).

b) IN-GFP increases fluorescence as compared to CTRL-PE2 (n=3 biological replicates of mESC).

c) IN-P2A-PE2 increases the amount of prime editing close to 2x when compared to CTRL-P2A-PE2 (n=3 biological replicates of mESC).

d) RT-qPCR on stable 5pl-PE2-GFP and IN-PE2-GFP mESC cell lines. 5pl-PE2-GFP has 1.3-fold higher RNA expression in mESC than IN-PE2-GFP. This additional experiment suggests that the IN dipeptide does not increase transcription. (N=6 independent replicates)

Data and error bars in b, c, and d indicate the median and standard deviation of independent biological replicates.
