# Supplementary material for: Peptide fusion improves prime editing efficiency
Source: Nat Commun. 2022 Jun 18;13:3512. doi: 10.1038/s41467-022-31270-y (PMC9206660; doi:10.1038/s41467-022-31270-y)
Supplement: Supplementary file 2 — Reporting Summary [file 41467_2022_31270_MOESM2_ESM.pdf]

nature portfolio

Corresponding author(s): Richard Sherwood

Last updated by author(s): May 18 2022

Reporting Summary

Nature Portfolio wishes to improve the reproducibility of the work that we publish. This form provides structure for consistency and transparency in reporting. For further information on Nature Portfolio policies, see our Editorial Policies and the Editorial Policy Checklist.

Statistics

For all statistical analyses, confirm that the following items are present in the figure legend, table legend, main text, or Methods section.

n/a	Confirmed	
☐	☒	The exact sample size (n) for each experimental group/condition, given as a discrete number and unit of measurement
☐	☒	A statement on whether measurements were taken from distinct samples or whether the same sample was measured repeatedly
☐	☒	The statistical test(s) used AND whether they are one- or two-sided<br>*Only common tests should be described solely by name; describe more complex techniques in the Methods section.*
☒	☐	A description of all covariates tested
☐	☒	A description of any assumptions or corrections, such as tests of normality and adjustment for multiple comparisons
☐	☒	A full description of the statistical parameters including central tendency (e.g. means) or other basic estimates (e.g. regression coefficient) AND variation (e.g. standard deviation) or associated estimates of uncertainty (e.g. confidence intervals)
☐	☒	For null hypothesis testing, the test statistic (e.g. F, t, r) with confidence intervals, effect sizes, degrees of freedom and P value noted<br>*Give P values as exact values whenever suitable.*
☒	☐	For Bayesian analysis, information on the choice of priors and Markov chain Monte Carlo settings
☒	☐	For hierarchical and complex designs, identification of the appropriate level for tests and full reporting of outcomes
☐	☒	Estimates of effect sizes (e.g. Cohen's d, Pearson's r), indicating how they were calculated

Our web collection on statistics for biologists contains articles on many of the points above.

Software and code

Policy information about availability of computer code

Data collection	No software was used to collect the data.
Data analysis	Data analysis performed using python packages scipy v.1.4.1, pandas v.1.1.5, numpy v.1.17.3 python v3.2.6<br>Figures generated using matplotlib v.3.1.1, and seaborn v.0.9.0 and plotly v5.8.0<br>Code used data processing and analysis can be found at https://github.com/maxwshen/prime-peptide<br>Underlying data used in analysis is included in the supplementary material

For manuscripts utilizing custom algorithms or software that are central to the research but not yet described in published literature, software must be made available to editors and reviewers. We strongly encourage code deposition in a community repository (e.g. GitHub). See the Nature Portfolio guidelines for submitting code & software for further information.

Data

Policy information about availability of data

All manuscripts must include a data availability statement. This statement should provide the following information, where applicable:

- Accession codes, unique identifiers, or web links for publicly available datasets
- A description of any restrictions on data availability
- For clinical datasets or third party data, please ensure that the statement adheres to our policy

Data used to generate figures and statistics is provided in the supplementary material. Code used for processing files provided in supplement can be found in the public repository: https://github.com/maxwshen/prime-peptide

Field-specific reporting

Please select the one below that is the best fit for your research. If you are not sure, read the appropriate sections before making your selection.

☒ Life sciences ☐ Behavioural & social sciences ☐ Ecological, evolutionary & environmental sciences

For a reference copy of the document with all sections, see nature.com/documents/nr-reporting-summary-flat.pdf

Life sciences study design

All studies must disclose on these points even when the disclosure is negative.

Sample size	Samples size for screen was always greater than 3 independant biological replicates, sufficiant in mammalian cell gene editing experiments to yield reproducible mean result values.
Data exclusions	No data has been excluded from this study
Replication	Minimum two experimental replicates were carried out for each set of treatment/control samples. All atempts of replication were successful
Randomization	Randomization is nor relavent to this study. All independant biological repilates were treated identically and all data was included.
Blinding	Mammalian cells used in this study were grown in identical conditions, no blinding has been used.

Reporting for specific materials, systems and methods

We require information from authors about some types of materials, experimental systems and methods used in many studies. Here, indicate whether each material, system or method listed is relevant to your study. If you are not sure if a list item applies to your research, read the appropriate section before selecting a response.

Materials & experimental systems

n/a	Involved in the study	
☒	☐	Antibodies
☐	☒	Eukaryotic cell lines
☒	☐	Palaeontology and archaeology
☒	☐	Animals and other organisms
☒	☐	Human research participants
☒	☐	Clinical data
☒	☐	Dual use research of concern

Methods

n/a	Involved in the study	
☒	☐	ChIP-seq
☐	☒	Flow cytometry
☒	☐	MRI-based neuroimaging

Eukaryotic cell lines

Policy information about cell lines

Cell line source(s)	All human cell lines were obtained from ATCC, U2OS, HCT116, HEK293FT and A549. Mouse embryonic cell lines were originally obtained as a gift from Hynek Wichterle at Columbia University, and were derived from Michael Kyba's lab originally
Authentication	Human cell lines were not authenticated since they were obtained from ATCC. Mouse embryonic cell lines were authenticated by RNA-seq profiling.
Mycoplasma contamination	All cell lines tested negative for mycoplasma contamination
Commonly misidentified lines (See ICLAC register)	No cell lines from ICLAC register have been used for this study.

Flow Cytometry

Plots

Confirm that:

☒ The axis labels state the marker and fluorochrome used (e.g. CD4-FITC).

☒ The axis scales are clearly visible. Include numbers along axes only for bottom left plot of group (a 'group' is an analysis of identical markers).

☒ All plots are contour plots with outliers or pseudocolor plots.

☒ A numerical value for number of cells or percentage (with statistics) is provided.

Methodology

Sample preparation	Cell lines were trypsinized, quenched, filtered through 0.22 um filter, and run through the flow cytometer.
Instrument	BD Biosciences FACS symphony
Software	FCS express 7 research
Cell population abundance	No cell sorting was performed. NA
Gating strategy	Cells were first gated via FSC-A and SSC-A to defined main population. Cells were next gated via SSC-A and SSC-W to define single cells. Plots in figures show all cells that pass these gates.

☒ Tick this box to confirm that a figure exemplifying the gating strategy is provided in the Supplementary Information.
