# Supplementary material for: Peptide fusion improves prime editing efficiency
Source: Nat Commun. 2022 Jun 18;13:3512. doi: 10.1038/s41467-022-31270-y (PMC9206660; doi:10.1038/s41467-022-31270-y)
Supplement: Supplementary file 12 — Description of Additional Supplementary Files [file 41467_2022_31270_MOESM12_ESM.pdf]

Title: Supplementary Data 1:
Description: 12,000 peptides used in PepSEq screens.

Title: Supplementary Data 2:
Description: Raw and processed data for the 12,000-peptide PepSEq screen. Paired Student's two-tailed t tests were performed to calculate p values.

Title: Supplementary Data 3:
Description: Raw and processed data for the 115-peptide PepSEq screen. Paired Student's two-tailed t tests were performed to calculate p values.

Title: Supplementary Data 4:
Description: Raw and processed data for the 100-dual-peptide PepSEq screen. Paired Student's two-tailed t tests were performed to calculate p values.

Title: Supplementary Data 5:
Description: Raw and processed data for the 100 pegRNA-target library experiments. Paired Student's two-tailed t tests were performed to calculate p values.

Title: Supplementary Data 6:
Description: List of primers used

Title: Supplementary Data 7:
Description: Raw and processed data for endogenous loci editing, NF2 and TP53.

Title: Supplementary Data 8:
Description: FACS analysis and gating statistics of CTRL-GFP-PE2 vs IN-GFP-PE2
